# A longitudinal and cross-sectional examination of the relationship between reasons for choosing a neighbourhood, physical activity and body mass index

**DOI:** 10.1186/1479-5868-7-57

**Published:** 2010-07-05

**Authors:** Tanya R Berry, John C Spence, Chris M Blanchard, Nicoleta Cutumisu, Joy Edwards, Genevieve Selfridge

**Affiliations:** 1 Faculty of Physical Education and Recreation, University of Alberta, Edmonton, Alberta, Canada; 2 Department of Medicine, Centre for Clinical Research Dalhousie University, Halifax, Nova Scotia, Canada; 3 Population and Public Health Portfolio, Alberta Health Services, Edmonton, Alberta, Canada

## Abstract

**Background:**

The purpose of this study was to examine the relationship between body mass index and neighborhood walkability, socioeconomic status (SES), reasons for choosing neighborhoods, physical activity, fruit and vegetable intake, and demographic variables.

**Methods:**

Two studies, one longitudinal and one cross-sectional, were conducted. Participants included adults (n = 572) who provided complete data in 2002 and 2008 and a concurrent sample from 2008 (n = 1164). Data were collected with longitudinal and cross-sectional telephone surveys. Objective measures of neighborhood characteristics (walkability and SES) were calculated using census data and geographic information.

**Results:**

In the longitudinal study, neighborhood choice for ease of walking and proximity to outdoor recreation interacted with whether participants had moved during the course of study to predict change in BMI over 6 years. Age, change in activity status, and neighborhood SES were also significant predictors of BMI change. Cross-sectionally, neighborhood SES and neighborhood choice for ease of walking were significantly related to BMI as were gender, age, activity level and fruit and vegetable intake.

**Conclusions:**

Results demonstrate that placing importance on choosing neighborhoods that are considered to be easily walkable is an important contributor to body weight. Findings that objectively measured neighbourhood SES and neighborhood choice variables contributed to BMI suggest that future research consider the role of neighborhood choice in examining the relationships between the built environment and body weight.

## Background

Obesity is a contributory factor in chronic diseases [1,2] and may decrease life expectancy by seven years [3]. Change in body mass index (BMI) over time is related to increased risk of disease. For example, gaining more than two BMI points over eight years increased risk of having a major cardiovascular event in men younger than sixty years [4] and BMI gains over fourteen years increased cardiovascular disease risk factors [1]. One area of research interest is the built environment because it is thought that aspects of urban form can contribute to decreased energy expenditure by limiting opportunities for walking [5]. However, despite evidence of a relationship between the walkability of a neighbourhood and change in body mass index (BMI) [6-9], questions remain as to the role that neighbourhood self-selection plays in this relationship [10].

The most popular index of objective walkability assesses density, diversity, design, and area in retail use [11]. Although there is some evidence that such a measure of walkability is associated with physical activity [9,12,13] and obesity [6-9], the majority of the evidence is cross-sectional and several longitudinal studies have called these relationships into question. Cross-sectional research indicates that children and adults who live in higher density, mixed-use neighbourhoods have lower rates of obesity than do people who live in lower density, residential-only neighbourhoods [9-12]. In one study, an improvement in the objective walkability of a neighbourhood of only 5% was associated with a 0.23 point reduction in BMI [14]. Similar longitudinal studies have resulted in mixed findings. Berry et al. [15] found no relationship between objectively measured walkability and change in BMI over six years. Lee et al. [16] reported a cross-sectional but not a longitudinal relationship between less urban sprawl and lower rates of obesity. They argue that cross-sectional data supporting relationships between walkable neighbourhoods and activity rates or obesity reflect self-selection to neighbourhoods rather than features within neighbourhoods influencing physical activity.

Indeed, Boone-Heinonen et al. [10] identified neighbourhood self-selection as the greatest limiter of existing research examining the relationship between the built environment and physical activity. Frank et al. [17] reported both neighbourhood preferences and actual neighbourhood characteristics influenced walking and driving choices. Those who preferred a highly walkable neighbourhood and lived in one walked the most and were the least likely to be obese. Participants who preferred a low walkable neighbourhood were more likely to be obese, regardless of what type of neighbourhood they actually lived in. Others found neighbourhood self-selection moderated the relationship between walkability and overall weekly minutes of walking, but not walking for transportation reasons [18].

Neighbourhood socioeconomic status (SES) may also predict BMI [15,19]. However, the relationship between built environment features and BMI has been shown to exist in high income communities but not in disadvantaged communities [20]. Lopez and Hynes [21] have referred to low rates of physical activity and high rates of obesity in low income urban areas that are objectively walkable as the inner-city paradox. They argue that this conflicting relationship is likely due to a mix of land-use, social, and infrastructure issues. For example poverty (social), abandoned buildings (land-use), and lack of adequate street lighting (infrastructure) combine to make walking a challenge for residents. Perceptions of the built environment such as believing one's neighbourhood has poor sidewalks, high crime rates, and physical disorder are also associated with higher levels of obesity [22-25].

The purpose of this research was to examine the role of reasons for choosing a neighbourhood in the relationship between the objectively measured built environment and obesity. The results of two studies are reported. The first study was a six-year longitudinal investigation of the relationship between objectively measured neighbourhood walkability and SES and change in BMI. Reasons for choosing a neighbourhood, whether participants had moved during the course of the study, and the interaction terms between neighbourhood choice and moving status were included in the model. The second study was cross-sectional research which also examined the relationship between objectively measured neighbourhood walkability and SES, and reasons for choosing a neighbourhood, and BMI. The cross-sectional survey was administered concurrent to the follow-up longitudinal study to a different sample of participants. Demographic factors such as age, gender, marital status and education [26,27], physical activity [28], and fruit and vegetable consumption [29] are all related to body weight. Therefore, these variables were also considered in the models to determine if the built environment and neighbourhood choice contributed to BMI beyond these already known factors.

## Methods

Data were collected through two studies: 1) a cross-sectional survey in 2008 and 2) a longitudinal study which consisted of two population surveys in 2002 and 2008 in the Edmonton region of Alberta. The survey target population for both studies was individuals aged 18 years or older and living in the former Capital Health region. The study populations were independent, with no overlap in participants between the two studies. The 2002 data from the longitudinal study were part of a population health survey conducted by the Population Health and Research department, Public Health, in the former Capital Health region, Edmonton. The purpose of the survey was to assess and monitor selected population health issues, health determinants, risk factors, and priorities in the Capital Health region (which includes the City of Edmonton and outlying regions). The cross-sectional survey was developed to be administered concurrently with the follow-up survey from the longitudinal study with the aim of asking questions that were not asked in 2002 and starting a new research cohort.

## Participants

There were 4175 participants in the 2002 sample of whom 3174 lived within the City of Edmonton. Of these participants, 3105 agreed to be contacted for future studies. The 2008 sample was restricted to those living within the City of Edmonton limits (n = 2362) since the geographic data layers that were used to calculate a walkability index are available for within city limits only. In 2008, 822 participants completed the follow-up survey. Of these participants, 222 had moved between 2002 and 2008 and 600 participants had not moved. Data from the original sample were gathered between October 28^th ^and December 15^th^, 2002. The follow-up survey took place between November 10, 2008 and January 15, 2009. The cross-sectional survey was completed by 1505 participants. Cross-sectional data were gathered between November 7^th^, 2008 and January 21^st^, 2009. After deletion of missing data, our final analysis included longitudinal data for 572 participants (435 nonmovers and 137 movers) and cross-sectional data for 1164 participants. According to Green [30], for a medium effect size in a regression model with 15 predictors, a sample size of 139 participants is needed. Thus, both our models were adequately powered.

## Measures

The data reported are based on measures that were identical between both studies. For comparability to other Canadian and provincial studies, most survey questions were consistent with the International Physical Activity Questionnaire (IPAQ) [31], and the Canadian Community Health Survey (CCHS) [32].

### Body Mass Index

Self-reported height and weight were used to estimate BMI. Participants in the longitudinal study reported their height and weight in 2002 and 2008. The change in BMI was calculated by subtracting the BMI in 2002 from the BMI in 2008.

### Sociodemographic variables

Socio-demographic questions were from the CCHS [32] and included age, gender, ethnicity, education, employment, marital status, and household annual income. In the 2008 longitudinal survey, participants were asked if they had moved since 2002.

### Fruit and vegetable consumption

Three questions based on the fruits and vegetables module of the CCHS [32] asked how many times per week participants usually drank fruit juices, and ate fruits and vegetables. A total weekly intake score was calculated and two groups were created: ate fewer than five servings of fruits and vegetables per day or ate five or more servings per day. This dichotomous variable was used in the cross-sectional analyses. In the longitudinal survey a change score was created by calculating which participants had changed groups. Participants characterized as stable did not change their fruit and vegetable consumption category, those participants who reported eating fewer than five servings per day in 2002 and reported consuming five or more servings in 2008 were classified as having increased consumption, and those who decreased were those who ate more than five servings per day in 2002 and fewer than five servings per day in 2008.

### Physical activity

Using the short-form of the IPAQ [31], participants were asked to recall how many minutes of walking, moderate and vigorous activity, sitting and sleeping they did over the last seven days. Total MET-minutes were calculated according to criteria set forth by the IPAQ research committee [33] and used to categorize participants as low, moderately, or highly active. The IPAQ has been shown to have adequate reliability and validity [34]. Stable, increased, and decreased categories were created in the same way as the fruit and vegetable change categories. To maintain adequate sample sizes within groups, only three change groups were created (e.g., participants categorized as "increased" may have gone from low activity in 2002 to either moderate or high activity groups in 2008).

### Neighbourhood Choice

Responses to ten statements were used to assess participants' possible reasons for choosing their neighbourhood [17]. Specifically, participants rated the importance of low crime, affordability, closeness to job, near shops and services, near major roads, ease of walking, low transportation costs, near outdoor recreation, quality of schools, and near to public transit on a 5 point scale: 1 (not at all important), 2 (of little importance), 3 (moderately important), 4 (of some importance) and 5 (very important) to neighbourhood choice. Each item was considered as an independent variable.

### Neighbourhood socioeconomic status (SES)

The description of how this index was developed is described in detail elsewhere [15]. In brief, neighbourhood SES indices were created from 2006 Canadian census data [35]. Participants provided postal codes which were assigned spatial reference and neighbourhood indices were created. Neighbourhoods were classified into high, medium, or low SES.

### Neighbourhood walkability

The description of how this index was developed is described in detail elsewhere [15]. Based on postal codes provided by participants, walkability indices were created from 2006 census data and from a taxation database provided by the City of Edmonton. Neighbourhoods were classified into very high, high, medium, very low, or low walkability.

### Data Analysis

Missing value analysis in SPSS was performed on BMI in both data sets and showed that it was missing at random. The EM algorithm multiple imputation procedure in SPSS was then used to impute the missing values. Objective neighbourhood walkability calculations involve an assessment of the number of dwellings per area in residential use. Census data provides the number of dwellings in each neighbourhood. Census data was compiled by the City of Edmonton at the neighbourhood level for 222 out of 340 neighbourhoods, with these 222 neighbourhoods being residential in character and the rest being mainly non-residential. As a result, information on neighbourhood walkability and SES was restricted to a subset of neighbourhoods only. Therefore, cases with missing data on these variables were removed.

Demographic variables were examined from each dataset to determine sample representativeness. The main analyses consisted of two linear regression models with BMI (cross-sectional) and BMI-change (longitudinal) as the criterion variables. Prior to conducting the main analyses, variables were selected according to various criteria for inclusion in the model. First, crosstabs were used to test whether any cells were empty or very small. We then tested whether the remaining neighbourhood choice variables were related to BMI change (longitudinal) or BMI (cross-sectional) through bivariate correlations. We then assessed the regression model for fit and excluded variables with poor fit before constructing the final model. Ethnicity was excluded from all models because an open-ended question generated 35 different responses (e.g., Canadian, Aboriginal, English) with many participants self-identifying as Canadian (longitudinal: 44.1%, cross-sectional: 38.7%). However this response could include Canadians of multiple racial backgrounds and thus was not informative. Data for those participants from the longitudinal sample who did not move are reported elsewhere [15] but it should be noted that the neighbourhood choice variables were not included in that analysis.

## Results

### Longitudinal analysis

To determine sample representativeness, demographic data from longitudinal participants were compared with the general population of Alberta. The longitudinal sample, in comparison to Albertans in general, was more highly educated (28.5% of the sample have a high school degree or less, in comparison to approximately 50% among Albertans), more likely to be married (66.4% reported being married or in a common-law partnership, in comparison to about 47% among Albertans) and marginally less likely to be employed (65.4% in comparison to 69.4% among Albertans) [36,37].

Choosing a neighbourhood due to affordability was unevenly distributed with 69.2% of participants rating this as important or very important. It was therefore not included in the regression model. The neighbourhood choice variables related to BMI-change, and included in the final regression model, were ease of walking, being close to outdoor recreation and quality of schools. The demographic variables of marital status, job status, and education status were not significant predictors of BMI change (all *p*'s > .336 in Step 1, Beta < .3 or > -.04). Age group and gender from 2002 were entered in the longitudinal model. We also included moving status (moved or not moved) as a possible moderating variable and tested the interaction of moving status with the neighbourhood choice variables according to procedures outlined in Aiken and West [38]. If necessary, follow-up tests of moderation were conducted using simple slopes procedures and the modgraph-I programme [39].

**Figure 1:**
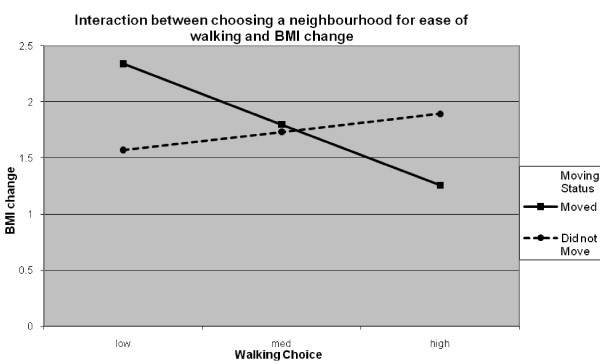
**Simple slopes graph showing the moderating effect of having moved or not and choosing a neighbourhood for ease of walking on change in BMI**.

**Figure 2:**
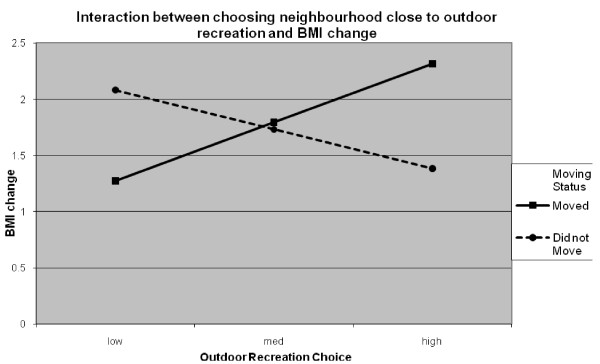
**Simple slopes graph showing the moderating effect of having moved or not and choosing a neighbourhood to be close to outdoor recreation on change in BMI**.

Collinearity was not a problem in the final longitudinal model with no variance inflation factor (VIF) value >1.95 and tolerance values between 0.52 and 0.99. As shown in Table 1, age, change in activity status, choosing to be close to outdoor recreation, the interaction between moving status and outdoor recreation, the interaction between moving status and choosing a neighbourhood because of ease of walking, and neighbourhood SES were significant predictors of change in BMI. Table 2 shows the BMI values from 2002 and 2008 in addition to the change in BMI. Younger participants had greater increases in BMI than older participants. Participants who decreased or did not change the amount of physical activity they did had increases in BMI whereas those participants who increased their physical activity had a small decrease in BMI. Participants in the lowest SES neighbourhoods had the largest increases in BMI compared to those in medium SES neighbourhoods and high SES neighbourhoods. Whether participants moved or not moderated the effects of choosing a neighbourhood for ease of walking and being close to outdoor recreation. As shown in figure 1, the slope for participants who moved was significant (t = -2.22, *p *< .05) with participants who rated ease of walking as low priority showing greater increases in BMI over 6 years than participants who rated this variable as important. The slope for those participants who did not move was not significant (t = -.99 *p > *.30). The results for the interaction between participants who chose a neighbourhood to be close to outdoor recreation and moving status is shown in figure 2. The slope for participants who moved was significant (t = 2.00, *p *< .05). Participants who rated this variable as high importance had greater gains in BMI than did participants who rated this variable as low importance. The opposite was found for participants who did not move (t = -2.182, *p *< .05).

**Table 1:** Linear regression model of the change in BMI across 6 years

Step		R-square Δ	Beta	T-value	p-value
1		.039			
	Gender		.040	0.98	. 293
	Age		-.156	-3.69	.000
	Moved		.007	.156	.876
	Change in activity		-.104	-2.52	.012
	Change in fruit and vegetable intake		.042	1.02	.309

2		.028			
	Gender		.040	1.01	. 314
	Age		-.156	-3.65	.000
	Moved		.011	.256	.798
	Change in activity		-.110	-2.69	.007
	Change in fruit and vegetable intake		.048	0.87	.384
	Walking (choice)		.048	.871	.384
	Recreation (choice)		-.117	-2.07	.039
	School (choice)		-.075	-1.44	.151
	Walking X move interaction		-.132	-2.55	.011
	Recreation X move interaction		.134	2.52	.012

	School X move interaction		.049	.952	.342
	
3		.008			
	Gender		.029	0.70	. 483
	Age		-.146	-3.39	.001
	Moved		.017	.384	.701
	Change in activity		-.111	-2.72	.007
	Change in fruit and vegetable intake		.051	1.24	.215
	Walking (choice)		.057	1.02	.307
	Recreation (choice)		-.114	-1.99	.046
	School (choice)		-.073	-1.41	.160
	Walking X move interaction		-.125	-2.40	.017
	Recreation X move interaction		.136	2.57	.011
	School X move interaction		.044	.858	.392
	Neighbourhood SES		-.083	-1.94	.053
	Neighbourhood walkability		-.068	-1.56	.116

**Table 2:** Mean (SD) BMI change by predictor variables from the longitudinal model

*Longitudinal*		*N*	*BMI 2002 mean (SD)*	*BMI 2009 mean (SD)*	*BMI change mean (SD)*
Gender	Male	292	26.90 (4.45)	27.23 (4.53)	.33 (2.83)
	Female	280	26.00 (4.84)	26.58 (5.16)	.62 (2.92)
Age	<50 years	302	26.02 (4.89)	26.92 (5.12)	.89 (3.16)
	>= 50 years	270	26.92 (4.36)	26.91 (4.54)	-.002 (2.44)

Moved	Moved	137	26.13 (5.31)	26.82 (5.41)	.70 (3.25)
	Did not Move	435	26.55 (4.44)	26.94 (4.67)	.40 (2.74)

Activity Category Change	Decrease	113	26.31 (4.95)	27.05 (5.06)	.75 (2.63)
	Stable	319	26.45 (4.40)	27.06 (4.79)	.61 (2.78)
	Increase	140	26.56 (5.04)	26.47 (4.83)	-.09 (3.20)

Fruit & Vegetable Change	Decrease	86	25.65 (4.07)	25.81 (4.17)	.15 (3.29)
	Stable	395	26.60 (4.79)	27.10 (4.97)	.51 (2.86)
	Increase	91	26.58 (4.60)	27.14 (4.86)	.57 (2.49)

Neighbourhood SES	Low SES	157	26.60 (4.87)	27.35 (5.61)	.75 (2.82)
	Medium SES	197	26.81 (4.88)	27.30 (4.72)	.48 (2.88)
	High SES	218	26.01 (4.29)	26.25 (4.30)	.25 (2.90)

Neighbourhood Walkability	Lowest	93	25.86 (5.04)	26.65 (5.23)	.81 (3.00)
	Low	115	26.20 (3.85)	27.16 (4.02)	.94 (2.57)
	Mid	106	26.74 (4.33)	27.20 (4.45)	.46 (2.72)
	High	128	27.04 (4.81)	26.83 (4.52)	-.20 (3.13)
	Highest	130	26.26 (5.13)	26.73 (5.83)	.47 (2.80)

### Cross-sectional

To determine sample representativeness, demographic data from cross-sectional participants were compared with the general population of Alberta. The cross-sectional sample, compared with Albertans in general was more highly educated (25% of the sample have a high school degree or less in comparison to approximately 50% among Albertans), more likely to be married (58.2% reported being married or in a common-law partnership, in comparison to about 47% among Albertans), marginally less likely to be employed (66.4% reported employment in comparison to 69.4% among Albertans) and older (19.4% of our sample was 65 or older, in comparison to approximately 10.4% of Albertans). Participants in our sample also tended to earn more than Albertans in general (mean income of Albertans is $48 017 per year) [36,37,40,41].

The variables related to choosing a neighbourhood because of crime and affordability were unevenly distributed and not included in the model. Choosing neighbourhoods close to jobs, shops and recreational facilities, quality of schools, and ease of walking, were correlated with BMI and included in the final regression model. Income was not included in the cross-sectional model because of a large amount of missing data (36.6%).

Collinearity was not a problem in the cross-sectional model with no VIF value > 1.22 and tolerance values between 0.72 and 0.96. In this model (Table 3), variables significantly related to BMI included gender, age, activity, fruit and vegetable intake, ease of walking and neighbourhood SES. Table 4 shows the mean BMI values. Older participants had higher BMI than younger participants, men had higher BMI than women, the lowest average BMI was seen in the most active participants compared to those moderately or low active, and those who ate fewer fruits and vegetables had higher BMI than those who ate more. In addition, the more importance participants placed on ease of walking when choosing a neighbourhood the lower their BMIs. Participants in low SES neighbourhoods had higher BMI than participants in medium SES or high SES neighbourhoods.

**Table 3:** Cross-sectional regression model

Step		R-square Δ	Beta	T-value	p-value
1		.058			
	Gender		-.155	-5.23	.000
	Age		.134	4.44	.000
	Marital status		.025	.833	.405
	Employment status		-.001	-.024	.981
	Education		-.001	-.030	.976
	Activity		-.074	-.2.51	.012
	Fruit and vegetable intake		-.073	-2.50	.012

2		.010			
	Gender		-.146	-4.92	.000
	Age		.130	4.23	.000
	Marital status		.036	1.20	.230
	Employment status		.007	0.22	.822
	Education		-.007	-0.25	.801
	Activity		-.066	-2.25	.028
	Fruit and vegetable intake		-.066	-2.21	.025
	Close to job		-.042	-1.37	.170
	Close to outdoor recreation		-.009	-0.28	.778
	Quality of schools		-.029	-0.94	.350
	Ease of walking		-.029	-0.93	.035

3		.009			
	Gender		-.146	-4.94	.000
	Age		.135	4.41	.000
	Marital status		.042	1.40	.161
	Employment status		.011	0.36	.717
	Education		.006	0.20	.838
	Activity		-.068	-2.28	.023
	Fruit and vegetable intake		-.059	-2.03	.043
	Close to job		-.043	-1.39	.164
	Close to outdoor recreation		-.010	-0.30	.765
	Quality of schools		-.027	-0.86	.393
	Ease of walking		-.061	-1.93	.057
	Neighbourhood walkability		-.051	-1.69	.091
	Neighbourhood SES		-.097	-3.18	.002

**Table 4:** Mean (SD) BMI by predictor variables in the cross-sectional model

*Cross-sectional*		*N*	*BMI mean (SD)*
Gender	Male	582	27.25 (5.41)
	Female	582	25.49 (5.54)
Age	<50 years	658	25.69 (5.13)
	>= 50 years	506	27.26 (5.92)
Job Status	Employed	814	26.26 (5.36)
	Other	350	26.62 (5.94)
Education	<= High School	260	26.46 (5.00)
	>High School	904	26.35 (5.69)

Activity Category	Low	235	27.28 (5.82)
	Moderate	543	26.29 (5.67)
	High	386	25.93 (5.12)

Fruit & Vegetable Intake	<5 servings/day	598	26.90 (5.76)
	>= 5 servings/day	566	25.81 (5.25)

Close to job	Not at all important	241	27.25 (5.76)
	Of little importance	160	26.37 (5.00)
	Moderately important	245	26.76 (5.88)
	Of some importance	237	26.00 (5.30)
	Very important	281	25.60 (5.44)

Close to outdoor recreation	Not at all important	165	27.17 (5.99)
	Of little importance	147	26.83 (4.87)
	Moderately important	286	26.15 (5.65)
	Of some importance	311	26.55 (5.66)
	Very important	255	25.62 (5.30)

Quality of schools	Not at all important	342	26.99 (5.84)
	Of little importance	106	26.18 (5.30)
	Moderately important	103	26.07 (5.33)
	Of some importance	192	25.64 (4.59)
	Very important	414	26.28 (5.79)

Ease of walking	Not at all important	95	27.49 (6.60)
	Of little importance	109	26.59 (4.58)
	Moderately important	262	26.79 (5.84)
	Of some importance	367	26.27 (5.68)
	Very important	330	25.76 (5.04)

Neighbourhood Walkability	Lowest	169	27.10 (5.23)
	Low	240	26.14 (5.41)
	Mid	210	26.58 (6.23)
	High	246	26.58 (5.75)
	Highest	299	25.83 (5.09)

Neighbourhood SES	Low SES	314	27.12 (5.93)
	Medium SES	371	26.07 (5.42)
	High SES	479	26.11 (5.34)

## Discussion

This research showed that age (in both models) and gender (in the cross-sectional model) were the variables most strongly related to BMI. However, the importance people place on reasons for choosing their neighbourhoods were also related to BMI. In particular, choosing neighbourhoods that make walking easier was associated with lower BMI in the cross-sectional model. This variable had as strong a relationship with BMI as other variables that are known to contribute to weight such as physical activity [28]. In the longitudinal model the interaction between moving status and the importance of choosing a neighbourhood for ease of walking was more strongly related to BMI than was change in physical activity. Specifically, participants who moved and rated ease of walking as not important had larger increases in BMI compared to those participants who moved and rated ease of walking as important. For those participants who did not move, there was not a significant change. These results reflect qualitative research in which developers reported believing that objectively measured walkable neighbourhoods attract walkers and if you are not interested in walking a walkable neighbourhood will not make a difference [42]. These findings are also similar to the cross-sectional findings of Frank et al. [17] who reported people who preferred a highly walkable neighbourhood were the least likely to be obese whereas those who preferred low walkable neighbourhoods were more likely to be obese. Thus, research should further investigate the characteristics of people who move to walkable neighbourhoods. Is it a case of people choosing neighbourhoods that fit with already held values or that features within the built environment can influence the behaviour of those previously not interested in walking?

This question is important given that objectively measured walkability was not a significant influence on BMI in either of our models, contradicting the findings of some researchers [5-8] while supporting a growing body of longitudinal evidence that has not supported the relationship between neighbourhood walkability and obesity [15]. However, neighbourhood walkability may influence physical activity [11,12] and this may occur despite no change in weight. Therefore, more longitudinal research is needed that examines the relationship between objectively measured neighbourhood walkability and change in physical activity behaviour, while controlling for questions of choosing a neighbourhood because it is perceived to be walkable.

Being close to outdoor recreation facilities was the other neighbourhood choice variable significantly related to BMI change but the results were mixed. For participants who moved, the more participants indicated that choosing a neighbourhood to be close to outdoor recreation facilities was important, the greater their BMI increased over the six years. The opposite relationship was found for participants who did not move and this variable was not related to BMI in the cross-sectional model. The longitudinal findings for movers are similar to those of Boehmer et al. [25] who reported lower odds of obesity were related to the perception of having fewer recreational facilities nearby (including both indoor and outdoor facilities such as trails, parks, and recreation centres). Future research should examine this relationship as it may reflect the "urban paradox" outlined by Lopez and Hynes [21]. That is, in lower SES neighbourhoods using outdoor recreation facilities such as parks may be considered risky or unappealing.

It is also important to consider that people who live in lower SES neighbourhoods likely have less ability to choose their ideal neighbourhood. It is also possible that our participants chose to live near outdoor recreation facilities for the benefit of other family members (e.g., children) rather than for themselves. It is also possible that people drive to outdoor recreation facilities and are not particularly active once they are there. Clearly, more research is needed to disentangle these findings. Indeed, the issue of neighbourhood SES is important to address as our findings support the conclusions of a systematic review that reported neighbourhood SES was related to obesity [43]. Our findings further contribute to the growing body of longitudinal evidence [15,19] showing low SES is associated with increased likelihood of being overweight. Although more research is needed to fully understand how neighbourhood SES contributes to obesity, it is without question that individuals in socially disadvantaged neighbourhoods face more barriers to health than their wealthier counterparts.

Another important variable that emerged was activity status. In both models, activity status was related to BMI. In the longitudinal model, those participants who increased their activity levels over six years showed a decrease in BMI. In the cross-sectional model, more activity was correlated with lower BMI. These findings are consistent with other research [28]. Creating more opportunities to be active can help problems of obesity. Our measure of activity did not capture activity related to walking, and in particular, walking for transportation or recreation. This may help to explain why we found no influence of neighbourhood walkability on BMI. Another potentially important variable in the weight equation, fruit and vegetable intake, significantly predicted BMI in the cross-sectional but not the longitudinal model. The results from our cross-sectional study replicate similar studies [29]. It may be that the findings were not significant in the longitudinal study, due to the short one week measure six years apart.

The inclusion of two separate studies, one of them longitudinal, is a strength of this research, providing a greater breadth of evidence. However, there are some limitations that should be noted. First, BMI was measured by self-report. Given that people tend to under report their actual weight [44], self-reported BMI usually provides an under representation of overweight and obesity at a population level. However, we have no reason to believe this bias differentially affected our findings. It should also be noted that the changes in BMI over 6 years were relatively small (i.e., less one BMI point) but the trends found in this research should be considered and examined in research that takes place over a longer period of time given the implications greater body weight have for health. Another limitation is the measures of physical activity and fruit and vegetable consumption, both of which ask participants to recall their behaviour over one week. This was particularly problematic in the longitudinal model where behaviour across one week was measured six years apart. An additional limitation is that we do not know when our participants moved in the longitudinal study. This is an important factor to consider in relation to BMI change. Further specificity of when participants moved would be of benefit and future research should take the recency of the move into account.

## Conclusions

Our studies provide evidence that choosing a neighbourhood based on certain features are important contributors to the relationship between the built environment and health outcomes such as body weight. Similar to Frank et al. [17], our findings showed that neighbourhood choice variables are associated with BMI. Thus, future research should follow the advice of Boone-Heinonen et al. [10] and consider the role of neighbourhood choice in examining the relationships between variables such as neighbourhood walkability and physical activity or body weight. Future research should also attempt to disentangle the relationship between neighbourhood SES and choosing neighbourhoods because of certain features. Although not examined in our research, it is very likely that people with higher SES have greater choice of neighbourhoods. This might help explain the "urban paradox" of inactive and overweight people living in lower income yet highly walkable neighbourhoods. It may be that those who are able to choose a walkable neighbourhood and want a walkable neighbourhood are those who are most influenced by positive design features.

## Competing interests

The authors declare that they have no competing interests.

## Authors' contributions

TRB, JCS, JE and CMB all contributed to research design. TRB wrote the first draft of the manuscript, and did the majority of the data analysis with CMB. NC developed the neighbourhood walkability and SES indices. GS contributed to manuscript writing and data analysis. All authors read and approved the final manuscript.
